# The Allergy Crossroads of Subtropical Regions: Mites, Crustaceans, and the Rise of Edible Insects

**DOI:** 10.3390/nu17091405

**Published:** 2025-04-22

**Authors:** Ruperto González-Pérez, Paloma Poza-Guedes, Manuel Alberto Figueiras-Rincón, Mónica Colque-Bayona, Inmaculada Sánchez-Machín

**Affiliations:** 1 Allergy Department, Hospital Universitario de Canarias, 38320 Santa Cruz de Tenerife, Spain; pozagdes@hotmail.com (P.P.-G.); manuelfigueiras@gmail.com (M.A.F.-R.); zerupean67@gmail.com (I.S.-M.); 2 Severe Asthma Unit, Hospital Universitario de Canarias, 38320 Santa Cruz de Tenerife, Spain; 3 Instituto de Investigación Sanitaria de Canarias (IISC), 38320 Santa Cruz de Tenerife, Spain; 4 Allergy Department, Hospital Universitario La Paz, 28046 Madrid, Spain; monica.colque.bayona@gmail.com; 5 Allergen Immunotherapy Unit, Hospital Universitario de Canarias, 38320 Santa Cruz de Tenerife, Spain

**Keywords:** novel foods, food safety, edible insects, mites, pan-allergens, component-resolved diagnostics, cross-reactivity

## Abstract

**Background:** Edible insects (EIs) are increasingly recognized as a sustainable protein source, yet concerns persist regarding allergic reactions, even in individuals without prior known consumption. This study examines the immune response profile in patients from a subtropical area to improve understanding of mite-related cross-reactivity and emerging food sensitizations. **Methods:** To assess sensitization to edible insects, we analyzed 634 patients from a tertiary care allergy institution with high perennial exposure to house dust mites and storage mites. Sensitization patterns were assessed using the ALEX²^®^ MacroArray platform, a multiplex IgE diagnostic tool covering 282 allergens, including *Locusta migratoria* (Lm), *Acheta domesticus* (Ad), and *T. molitor* (Tm). Patients with IgE levels ≥0.3 kU/L were evaluated for cross-reactivity to both mite allergens and pan-allergens. **Results**: Of the 634 patients, 138 (21.76%) exhibited IgE sensitization to at least one EI extract. Tropomyosin was the most prevalent pan-allergen (63.76%), followed by troponin-C (28.98%) and arginine kinase (26.81%). Notably, 95.66% of EI-sensitized individuals also reacted to mite allergens. However, 23.18% lacked reactivity to common pan-allergens, suggesting alternative sensitization mechanisms. **Conclusions**: This investigation can highlight regional variations in EI sensitization, where high mite exposure in subtropical climates appears to influence IgE responses to insect proteins. The findings suggest that EI sensitization is not merely incidental but represents a distinct immunological phenomenon shaped by environmental factors and allergen cross-reactivity. Since the presence of food-specific IgE does not reliably indicate clinical allergy, and the lack of food challenge data constrains diagnostic certainty, acknowledging EI sensitization as a potential risk factor remains essential for ensuring food safety and protecting public health.

## 1. Introduction

On 20 January 2025, the European Commission approved UV-treated powder derived from *Tenebrio molitor* (Tm) larvae as a food ingredient for the general population under regulated conditions. The powder is produced from whole larvae through a series of processing steps including blanching, drying, and milling, resulting in a high-protein product suitable for incorporation into baked goods, snacks, pasta, and other food formulations [1]. This decision aligns with the growing interest in edible insects (EI) as a sustainable protein source, yet it also raises concerns regarding allergenicity. The European Food Safety Authority (EFSA) has highlighted potential risks, particularly for individuals with known pre-existing allergies to crustaceans, dust mites, mollusks, or components found in insect feed, mandating specific labeling requirements to mitigate these concerns and ensure consumer safety [2].

The allergenic potential of EIs is of particular interest due to their evolutionary proximity to crustaceans and Acari, both of which contain well-characterized allergens. Research has identified proteins such as tropomyosin (TM), α-amylase, and arginine kinase (AK) as major pan-allergens capable of eliciting immune responses. These proteins exhibit IgE-binding cross-reactivity with homologous allergens found in arthropods (e.g., mites, crustaceans), mollusks, and even certain nematodes [3,4]. As a result, individuals with shellfish allergies may be at risk of severe allergic reactions, including anaphylaxis, when consuming edible insects. While the link between EI consumption and crustacean allergies is well established, the impact of insect-derived allergens on individuals with mite allergies remains less clear, prompting further investigation [5,6].

The concept of mite-EI syndrome is gaining attention in allergology as an increasing number of individuals sensitized to house dust mites (HDMs) and storage mites report allergic reactions to insect-based foods [7,8]. Given the strong cross-reactivity between mites, crustaceans, and insects, ingestion of edible insect products could trigger symptoms ranging from mild oral allergy syndrome to life-threatening anaphylaxis [9,10]. Documented cases indicate that individuals with known HDM or crustacean allergies may later develop sensitization to mealworms, grasshoppers, or other edible insects, yet it remains uncertain whether mite sensitization alone is sufficient to induce a true food allergy [11,12]. The clinical significance of this cross-reactivity and its potential to contribute to allergic disease burden require further exploration [13].

Beyond individual sensitization patterns, the allergen exposome—comprising environmental factors such as climate, urbanization, dietary habits, microbiota composition, and exposure to pollutants—plays a crucial role in shaping immune responses [14,15]. Geographic variability influences specific IgE (sIgE) profiles, potentially affecting the prevalence and severity of edible insect allergies across different populations. Regions with high, year-round exposure to HDMs and storage mites may exhibit distinct sensitization patterns compared to areas where such exposure is less prevalent [16,17]. Furthermore, climate change is already altering the seasonality, production, concentration, allergenicity, and geographic spread of airborne allergens, with measurable consequences for human health [18,19]. Tenerife (28°16′7″ N, 16°36′20″ W), the largest and highest island of the Canary archipelago, exemplifies this interplay between climate and allergic disease. Its climate, shaped by the cool, humid northeast trade winds linked to the Azores anticyclone, generally results in lower air pollution levels than those observed in mainland Europe. Moreover, Tenerife’s climate classification (Köppen BWh) and varied elevations contribute to heterogeneous but continuous allergen exposure throughout the year [20,21].

Molecular allergy diagnostics constitute a pivotal advancement in the field of precision medicine, enabling the characterization of individual sensitization profiles with high specificity and resolution. This approach facilitates a tailored understanding of allergic diseases by identifying sIgE antibodies directed against discrete allergenic molecules [22,23]. Currently, molecular diagnostics are implemented through two principal methodologies: single-component assays, which quantify sIgE against individual allergen molecules, and multiplex assays, which allow for the simultaneous detection of sIgE to a broad array of allergenic components within a single analytical procedure [24,25].

Multiplex molecular diagnostics leverage sophisticated in vitro platforms that integrate allergen microarrays and nanotechnology to achieve high-throughput immunoassays. These technologies employ solid-phase substrates—such as glass, plastic, or cellulose—onto which defined arrays of allergenic molecules are immobilized. When incubated with a patient’s biological sample, specific IgE antibodies, if present, bind to their cognate allergens, forming immune complexes. These complexes are subsequently visualized and quantified using advanced immunochemical detection systems, including fluorescence, chemiluminescence, mass spectrometry, densitometry, or electrochemical readouts. This multiplex strategy is particularly valuable in the clinical evaluation of polysensitized individuals, allowing for comprehensive sensitization profiling using minimal sample volume. Among the available platforms, the Immuno Solid-phase Allergen Chip (ISAC; ThermoFisher Scientific, Waltham, MA, USA) and the Allergy Explorer (ALEX; Macroarray Diagnostics, Vienna, Austria) are the most widely implemented. While both platforms provide extensive allergen panels, they differ substantially in analytical scope, immunochemical methodology, technical workflow, result interpretation, and serum volume requirements [26]. Notably, the ISAC platform focuses exclusively on the quantification of sIgE to purified allergen components, whereas the ALEX system extends its utility by incorporating measurements of sIgE to both allergen components and whole extracts, in addition to total serum IgE, thereby offering a more integrative diagnostic output.

In this context, the present study aims to characterize the molecular profile of individuals sensitized to EIs despite no prior conscious exposure. Conducted within this subtropical region (Tenerife, Spain) with persistent HDM and storage mite exposure, yet relatively low rates of intestinal parasitic infections and cockroach sensitization, this research seeks to clarify the clinical relevance of mite-related cross-reactivity and contribute to a broader understanding of emerging food allergies associated with entomophagy [27,28].

## 2. Materials and Methods

### 2.1. Subjects

Between March and September 2024, we conducted a retrospective analysis of consecutive patients attending the Outpatient Allergy Clinic, Immunotherapy, and Severe Asthma Unit at Hospital Universitario de Canarias in Tenerife, Spain. Eligible participants were required to exhibit IgE-mediated reactivity, as determined by a molecular allergen diagnostic platform, and have a clinical history suggestive of allergy-mediated disease. Sensitization to at least one insect extract—migratory locust (*Locusta migratoria*, Lm), house cricket (*Acheta domesticus*, Ad), or mealworm (*Tenebrio molitor*, Tm)—as detected through IgE microarray analysis, defined the study subgroup for data evaluation. This investigation received approval from the local Ethical Committee (approval code CHUC 2023 66). Written informed consent was obtained from all participants, with parental or guardian consent required for individuals under 18 years of age.

**Figure 1:**
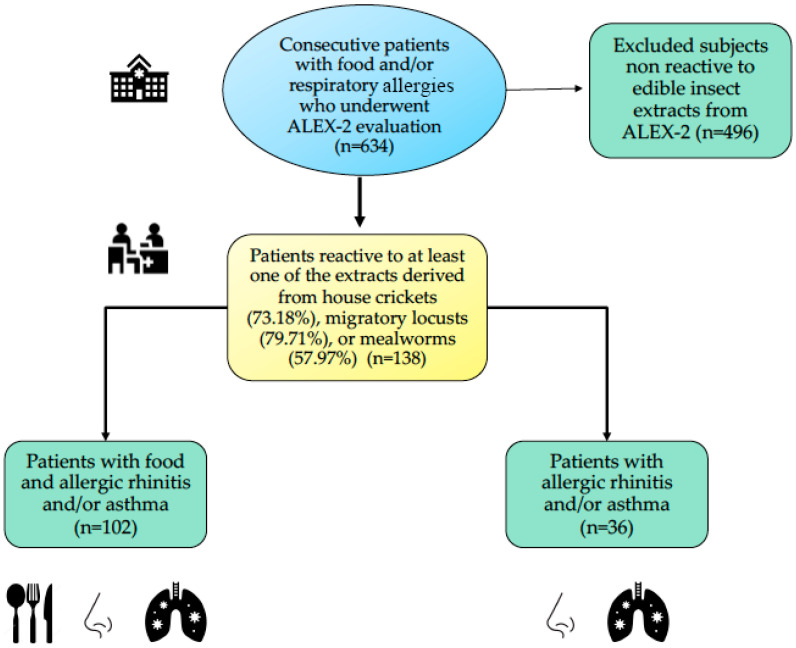
Flow diagram of patients and study selection. ALEX-2^®^: Allergy Explorer-2^®^ MacroArray platform (MacroArray Diagnostics, Vienna, Austria).

Clinical data were extracted from patients’ medical records, including sociodemographic information, clinical history—encompassing past medical conditions and current allergy diagnoses—and medication details. The severity and stage of allergic diseases were assessed following established guidelines [29,30]. Patients who had previously undergone or were currently receiving allergen immunotherapy or monoclonal antibody (biologic) treatment were excluded. No nutritional supplementations were administered as part of the study protocol. Additionally, pregnant and breastfeeding women were not included in the study (Figure 1).

### 2.2. Serological Analysis

Peripheral blood samples were obtained from all participants, and anonymized identification codes were assigned to ensure confidentiality and data traceability. Blood collection did not require participants to fast or discontinue ongoing medications, thereby minimizing disruption to their clinical management. Samples were stored at −40 °C until analysis and thawed immediately prior to in vitro testing. Quantification of specific IgE (sIgE) and total IgE (tIgE) was performed using the ALEX²^®^ MacroArray platform (MacroArray Diagnostics, Vienna, Austria), a standardized multiplex immunoassay conducted in accordance with the manufacturer’s protocol. The ALEX²^®^ platform enables comprehensive profiling of IgE sensitization by simultaneously detecting tIgE and sIgE against a panel of 295 allergenic targets, comprising 117 native allergen extracts and 178 molecular components. In the present study, 282 of these reagents were analyzed, including 157 whole extracts—among them *Ad*, *Lm*, and *Tm*—and 125 individual allergenic molecules [31]. Allergen components are covalently bound to functionalized polystyrene nanobeads and arrayed on a nitrocellulose membrane, facilitating high-density immobilization and optimal epitope presentation. This design enhances assay sensitivity and allows for efficient detection within a minimal matrix surface area [32,33].

The assay quantitatively measures sIgE concentrations in the range of 0.3–50 kUA/L and provides semi-quantitative tIgE values from 1 to 2500 kU/L. sIgE values ≥ 0.3 kUA/L were considered indicative of sensitization. Results were expressed both as absolute concentrations and in five categorical classes (0–4), reflecting increasing levels of IgE reactivity. To improve diagnostic specificity and minimize false-positive results due to non-clinically relevant IgE responses—particularly those arising from cross-reactive carbohydrate determinants (CCDs)—the assay incorporates a proprietary anti-CCD inhibitor. This feature is especially valuable in polysensitized individuals, where distinguishing clinically significant sensitizations is essential [34,35].

The array included 17 molecular allergens derived from mites, including Der p 1, Der p 2, Der p 5, Der p 7, Der p 10, Der p 11, Der p 20, Der p 21, Der p 23, Der f 1, Der f 2, Blo t 5, Blo t 10, Blo t 21, Lep d 2, Gly d 2, and Tyr p 2. Tropomyosin sensitization was defined by IgE reactivity to Ani s 3, Blo t 10, Der p 10, Pen m 1, or Per a 7. Sensitization to AKs was identified through reactivity to Pen m 2, Bla g 9, or Der p 20. Additional muscle-related markers included Pen m 3 (myosin light chain), Pen m 4 (sarcoplasmic calcium-binding protein), Cra c 6 (troponin-C), and Der p 11 (paramyosin). A minimum of 100 µL of serum or plasma (excluding EDTA plasma) was required for the assay, which had a total processing time of approximately 3.5 h.

### 2.3. Statistical Analysis

Demographic characteristics were summarized using medians and standard deviations for continuous variables and percentages for categorical variables. Group differences were analyzed using appropriate statistical tests: Analysis of Variance (ANOVA) was applied to parametric continuous variables, while the Kruskal–Wallis and Mann–Whitney U tests were used for nonparametric continuous variables. Categorical variables were compared using the Chi-square test. Statistical significance was set at a *p*-value < 0.05. To assess allergen associations, simple logistic regression was performed, adjusting for potential confounding variables. All statistical analyses were conducted using GraphPad Prism version 10.0.0 (GraphPad Software, La Jolla, CA, USA).

## 3. Results

### 3.1. Study Population

Between March and September 2024, a proteomic analysis was conducted using the ALEX²^®^ MacroArray platform on 634 consecutive patients referred for allergy assessment. Sensitization to at least one EI extract—Lm, Ad, and/or Tm—was identified in 138 individuals, accounting for 21.76% of the cohort.

The median age of sensitized individuals was 17 years (range: 3–75), with the majority being male (68.84%; 95/138). A clinical history indicative of food and/or respiratory allergy was reported in most cases. Specifically, 58.0% (80/138) of patients experienced both food and respiratory allergic symptoms, whereas 15.9% (22/138) reported food allergy alone. Among those reporting respiratory manifestations (36/138; 26.1%), 58.3% had allergic rhinitis and 41.6% had a combined diagnosis of allergic rhinitis and asthma. Notably, seafood allergy was documented in 50.0% (40/80) of individuals with food-related symptoms. Median total IgE levels were elevated in this group, reaching 532 IU/mL, with values ranging from 54 to 2500 IU/mL. A positive family history of atopy was reported in 77.08% (105/138) of the sensitized individuals (Table 1).

### 3.2. Specific IgE Profile in Patients with a Sensitization to EIs

Sensitization to at least one EI extract was identified in 138 individuals, with the following distribution: Lm in 110 individuals (79.71%), Ad in 101 (73.18%), and Tm in 80 (57.97%). Among the sensitized individuals, 65 (47.1%) exhibited concurrent sensitization to all three EIs. Single-reactor cases were distributed as follows: Lm = 22 (15.94%), Ad = 19 (13.76%), and Tm = 8 (5.79%).

Tropomyosin emerged as the serodominant allergen (63.76%) in our cohort, followed by troponin-C (28.98%), AK (26.81%), and sarcoplasmic calcium-binding protein (8.69%). Additionally, 6.52% of subjects were sensitized to myosin light chain (Pen m 3), while only one individual (0.72%) displayed IgE reactivity to Der p 11 paramyosin (Table 2).

Interestingly, 32 out of 138 individuals (23.18%) sensitized to EIs showed no IgE reactivity to any of the pan-allergens—TM, AK, paramyosin, troponin-C, and myosin light chain—on the ALEX2^®^ chip. Additionally, only 4 of the 138 subjects (2.89%) sensitized to EIs in the current study exhibited no reactivity to any of the molecules included in the microarray panel.

### 3.3. Multiplex IgE Reactivity Profiles in Patients with Sensitization to EIs and Exclusively Affected by Respiratory Allergies

All 36 insect-reactive individuals (100%) diagnosed with allergic rhinitis and/or asthma, but not food allergies, were sensitized to at least one mite allergen. Eight allergens—Der f 2, Der p 23, Der p 2, Der p 1, Der f 1, Der p 5, Der p 7, and Blo t 21—were identified in over 50% of the cohort, making them serodominant. The majority of patients were cross-sensitized to group 2 mite allergens, specifically Der f 2 and Der p 2, with lesser cross-sensitization to Gly d 2, Tyr p 2, and Lep d 2. Sensitization to storage mite group 2 allergens was notably high, dominated by Gly d 2 (47.22%), followed by Tyr p 2 (41.66%) and Lep d 2 (36.11%). Remarkably, reactivity to pan-allergens was infrequent (5 out of 36 patients), with Der p 20 in 3 cases (8.33%), Der p 10 in 1 case (2.77%), and Blo t 10 in 1 case (2.77%). No cases of Der p 11 sensitization were observed (Table 3).

### 3.4. Allergen-Specific IgE Levels to TMs, AKs, and Different EI Extracts Were Significantly Correlated

In the investigated cohort, only 6 out of 138 individuals (4.34%) sensitized to EIs showed no reactivity to any of the 17 mite allergens tested, which included Der p 1, Der p 2, Der p 5, Der p 7, Der p 10, Der p 11, Der p 20, Der p 21, Der p 23, Der f 1, Der f 2, Blo t 5, Blo t 10, Blo t 21, Lep d 2, Gly d 2, and Tyr p 2. Among these 17 mite-derived molecules, significant correlations with sensitization to EIs were found only for two allergen groups: AK with Der p 20 (r = 0.26) and TM with Blo t 10 (r = 0.78) and Der p 10 (r = 0.86) (Appendix A).

## 4. Discussion

The increasing use of EIs as a sustainable protein source has raised concerns about their allergenic potential [36,37]. Mite-EI syndrome exemplifies a complex interplay between insects, mites, and their environments, particularly in subtropical regions with high mite prevalence. This syndrome, a subset of the broader dust mite–crustacean–insect syndrome, underscores the significant cross-reactivity among these arthropods, often affecting individuals sensitized to mites who subsequently develop allergies to crustaceans and EIs [38,39]. Understanding these immune mechanisms provides valuable insights into allergen cross-reactivity beyond single food sources. Lipid transfer protein (LTP) syndrome, a leading cause of plant-derived food allergies, provides a useful parallel [40,41]. LTPs, as stable pan-allergens found in various plant species, often initiate sensitization through inhalant exposure before progressing to food allergies. Similarly, considering the strong correlations observed between EIs and mite sensitization, we hypothesize that in mite-prevalent regions, inhalant exposure to HDMs or storage mites may act as an initial sensitizing factor for EIs, even among individuals without prior direct contact. However, this proposed mechanism remains hypothetical, as the observational nature of the study precludes any inference of causality. It is therefore essential to interpret these associations as indicative rather than conclusive. Former research has demonstrated that shrimp allergies can be strictly dependent on HDM sensitization, a pattern that may extend to EI allergies in certain geographic areas [42,43]. Moreover, recent studies have pointed out the clinical relevance of sensitization to tropomyosin—a highly conserved pan-allergen present in crustaceans, mites, and insects—among allergic individuals in Europe. Specifically, sensitization to shrimp TM (e.g., Pen m 1) and its homologues in HDMs (e.g., Der p 10) and EIs has been reported with increasing frequency. A multicenter study in an Italian pediatric cohort demonstrated that tropomyosin sensitization was strongly associated with shrimp-induced anaphylaxis, even in individuals without prior shellfish exposure [44]. Similarly, evidence from the Netherlands suggests a substantial risk of cross-reactivity to edible insects in shrimp-allergic individuals due to shared allergenic proteins, particularly TM [45]. Furthermore, a recent paper examines regional differences in component-resolved diagnosis (CRD) practices and allergen sensitization patterns across the Asia-Pacific region [46]. The authors highlight the influence of environmental and genetic factors on CRD outcomes and emphasize the need for region-specific diagnostic panels and advocate for localized approaches and greater regional collaboration to improve diagnostic precision and allergy management. In parallel, a comparative study of cockroach-sensitized individuals from Asia and Central Europe revealed significant differences in IgE reactivity profiles, indicating variation in primary sensitizing allergen sources across regions [47]. These findings highlight the importance of considering regional sensitization patterns when evaluating emerging allergenic risks, such as those posed by novel foods or environmental exposures in mite-prevalent areas.

### 4.1. Molecular Sensitization Patterns in the Investigated Cohort

In the present study, TM was the most prevalent allergen (63.76%), followed by troponin-C (28.98%), AK (26.81%), and sarcoplasmic calcium-binding protein (8.69%) [31,32]. A smaller proportion (6.52%) were sensitized to myosin light chain (Pen m 3), and only one individual (0.72%) reacted to Der p 11 paramyosin. Notably, 23.18% of participants exhibited no IgE reactivity to the pan-allergens on the ALEX2^®^ chip. This contrasts with a Mediterranean cohort where 55.4% of insect-reactive individuals lacked pan-allergen sensitization, suggesting possible regional variations in sensitization profiles [48].

Several plausible explanations merit consideration. One possibility is sensitization to insect-specific proteins not currently represented in the ALEX2^®^ panel, pointing out the limitations inherent to predefined allergen arrays. Alternatively, these individuals may exhibit reactivity to pan-allergens at levels below the assay’s detection threshold, thereby eluding identification despite a biologically relevant response [49,50]. A further explanation involves the prospect of true primary sensitization to unique insect-derived allergens that lack significant structural homology with known cross-reactive molecules, pointing to a distinct immunological pathway. To explore these hypotheses, future investigations should consider the integration of high-sensitivity singleplex immunoassays, proteomic profiling, and experimental models capable of characterizing novel allergenic components [51,52]. Such approaches would not only enhance diagnostic precision but also contribute to a deeper understanding of the molecular complexity underlying insect allergen sensitization. Interestingly, a subset of four participants in our cohort displayed specific IgE to EIs but no reactivity to other food or inhalant allergens, suggesting that EIs may serve as a primary sensitizer in some individuals. These findings highlight the potential for EIs to be an independent cause of allergic sensitization, rather than solely a result of cross-reactivity.

### 4.2. Cross-Reactivity Among EIs and Other Allergens

This investigation confirms relevant cross-reactivity between EIs and crustaceans, driven by pan-allergens such as TM, troponin-C, and AK. However, the relationship between mite and EI sensitization remains debated. Previous research suggested an inverse correlation between mite sensitization and IgE reactivity to EIs, implying mites might not act as primary sensitizers [53,54]. Our findings challenge this assumption, showing that 95.66% of individuals sensitized to EIs also reacted to at least one of the 17 mite allergens investigated, with significant correlations between EI sensitization and key molecular pan-allergens. In this regard, despite former research having shown that individuals with HDM allergies may experience allergic reactions after consuming EIs, the clinical relevance of co-sensitization to mites and EIs is still debated, with more studies required to understand the mechanisms of allergic responses. Despite epidemiological data on allergic reactions to EIs among patients with mite allergies being scarce, in a recent study of 6173 individuals, 4.3% showed sensitization to yellow mealworm, with a notable association between this sensitization and HDM allergies [55]. In our cohort, among the 36 EI-reactive individuals diagnosed exclusively with allergic rhinitis and/or asthma (without food allergies), all were sensitized to at least one mite molecule. However, they exhibited infrequent reactivity to pan-allergens such as Der p 20 (8.33%), Der p 10 (2.77%), and Blo t 10 (2.77%). In addition, only 6 out of 138 individuals (4.34%) sensitized to EIs exhibited no reactivity to any of the 17 mite allergens assessed, which included Der p 1, Der p 2, Der p 5, Der p 7, Der p 10, Der p 11, Der p 20, Der p 21, Der p 23, Der f 1, Der f 2, Blo t 5, Blo t 10, Blo t 21, Lep d 2, Gly d 2, and Tyr p 2. Among these allergens, statistically significant correlations with sensitization to EIs were observed for two molecular groups: arginine kinase (AK) with Der p 20 (r = 0.26, *p* < 0.05) and TMs with Blo t 10 (r = 0.78, *p* < 0.05) and Der p 10 (r = 0.86, *p* < 0.05). While all three correlations reached statistical significance, the magnitude of association varied substantially. Specifically, the correlations involving TM were strong, suggesting a potentially meaningful immunological relationship with EI sensitization. In contrast, the correlation between AK and Der p 20 was weak, and although statistically significant, it may be of limited biological relevance. This distinction emphasizes the importance of considering both the statistical significance and the strength of associations when interpreting sensitization profiles, as stronger correlations are more likely to reflect clinically relevant patterns.

### 4.3. Insect-Specific Proteins and Sensitization Mechanisms

While cross-reactivity explains some EI sensitization cases, the presence of insect-specific proteins such as chemosensory proteins, odorant-binding proteins, and hexamerin suggests alternative sensitization pathways [56,57]. These proteins, largely absent in phylogenetically related organisms such as mites and crustaceans, may contribute independently to EI sensitization. In this context, the second installment of the Acari Hypothesis, “Interspecies Operability of Pattern Recognition Receptors,” offers a compelling framework for understanding atypical sensitization [58]. It proposes that pattern recognition receptors (PRRs) from acarians—such as fibrinogen-related proteins and ixoderins—bind to dietary molecules problematic to the mite. These PRR ligand complexes may enter the human body through inhalation, ingestion, or ectoparasitic contact and be misidentified as immunological threats, thereby triggering IgE-mediated responses [59,60]. This mechanism not only provides a plausible basis for allergen cross-reactivity but also offers a potential explanation for primary sensitization in the absence of known exposures. Together, these insights underscore the importance of addressing both diagnostic limitations and immunological complexity when evaluating novel allergenic sources such as EI. Further research is needed to elucidate the precise mechanisms involved.

### 4.4. Limitations

Diagnosing mite-EI syndrome is challenging due to overlapping allergens among mites, crustaceans, and EIs [61,62]. Although component-resolved diagnostics could help distinguish primary sensitization from cross-reactivity, their limited availability restricts precise assessments. Moreover, treatment options for this syndrome remain limited, with management largely focused on avoidance and emergency preparedness in case of anaphylaxis [63,64,65]. A major limitation of this investigation was the absence of clinical food challenges, which are crucial for assessing the clinical significance of insect-specific sensitization patterns. While food challenges have been used successfully in shrimp-allergic patients, particularly those co-sensitized to specific pan-allergens, similar investigations in mite-allergic populations are lacking [66,67,68]. Furthermore, the limited sample size of our cohort restricts the generalizability of the findings. In addition, the absence of clinical data for excluded patients precluded direct comparisons between included and excluded individuals.

## 5. Conclusions

Despite the growing recognition of the allergenic potential of edible insects, further research is urgently needed to clarify the clinical relevance of cross-reactivity between mites, crustaceans, and EIs. Public health policies should prioritize addressing the risks of allergic reactions to edible insects, particularly among individuals with known mite or shellfish allergies [69,70,71]. Regulatory frameworks should continue to include allergen labeling, consumer education, and further research into strategies for reducing the allergenicity of edible insects.

This investigation highlights regional variations in molecular sensitization profiles among individuals reactive to EIs. In subtropical regions, increased mite exposure appears to influence IgE responses to insect proteins, emphasizing the complex interplay between environmental factors and allergen cross-reactivity and suggesting that food sensitization is shaped by multiple determinants. Despite the lack of a definitive correlation between food-specific IgE and clinical allergies, the diagnostic process remains complex and necessitates a comprehensive medical history, detailed laboratory investigations, and, in many cases, oral food challenges [72,73]. Although the absence of specific food challenge data limits the clinical assessment of insect-specific sensitization in mite-allergic individuals, the present findings indicate the need to recognize EI sensitization as a distinct immunological concern rather than an incidental phenomenon.

## Figures and Tables

**Table 1:** Demographic and clinical characterization data of the investigated cohort.

Characteristics	(*n* = 138)
Age (y.o.) median (range)	17 (3–75)
Sex (F/M)	43/95
Food and respiratory allergy	80 (58%)
Food allergy	22 (15.9%)
Seafood allergy	40 (50%)
Respiratory allergy	36 (26%)
Allergic rhinitis	21 (58.3%)
Allergic rhinitis and asthma	15 (41.6%
Total IgE (IU/mL) median (range)	532 (54–2500)
Family history of atopy (%)	105 (77.08)

**Table 2:** Number (%) of selected patients (*n* = 138) with a sensitization to edible insect/s and IgE reactivity to pan-allergens. Tropomyosin reactivity involves IgE antibodies to Ani s 3, Blo t 10, Der p 10, Pen m 1, or Per a 7. Arginine kinase sensitization is defined by reactivity to Pen m 2, Bla g 9, or Der p 20. Additional markers include Pen m 3 (myosin light chain), Pen m 4 (calcium-binding protein), Cra c 6 (troponin-C), and Der p 11 (paramyosin). “None” indicates no IgE reactivity to any pan-allergens.

Pan-Allergens in 138 Subjects Sensitized to at Least One Edible Insect	*Acheta domesticus * (*n* = 101)	*Locusta migratoria * (*n* = 110)	*Tenebrio molitor * (*n* = 80)
Tropomyosin (any) molecules in 88/138 subjects (63.76%)			
Ani s 3 (*n* = 76)	69 (68.31)	67 (60.9)	58 (72.5)
Blo t 10 (*n* = 79)	71 (70.29)	68 (61.81)	62 (77.5)
Der p 10 (*n* = 64)	62 (61.38)	58 (52.72)	57 (71.25)
Per a 7 (*n* = 68)	64 (63.36)	60 (54.54)	58 (72.5)
Pen m 1 (*n* = 63)	62 (61.38)	58 (52.72)	54 (67.5)
Arginine kinase (any) molecules in 46/138 subjects (33.33%)			
Bla g 9 (*n* = 36)	27 (26.73)	33 (30)	22 (27.5)
Der p 20 (*n* = 39)	32 (31.68)	35 (31.81)	27 (33.75)
Pen m 2 (*n* = 26)	23 (22.77)	25 (22.72)	23 (28.75)
Paramyosin Der p 11 in 1/138 subjects (0.72%)	1 (0.99)	1 (0.9)	1 (1.25)
Troponin-C Cra c 6 in 40/138 subjects (28.98%)	34 (33.66)	37 (33.63)	31 (76.25)
Myosin light chain Pen m 3 in 9/138 subjects (6.52%)	8 (7.92)	8 (7.27)	8 (10)
Sarcoplasmic calcium-binding protein Pen m 4 in 12/138 subjects (8.69%)	8 (7.92)	10 (9.09)	8 (10)
None (32/138 (23.18%) subjects)	24 (23.76)	22 (20)	10 (10)

**Table 3:** Serological analysis of specific IgE (sIgE) responses (kU/L) to 17 mite molecular allergens in selected patients (*n* = 36) with a sensitization to at least one edible insect and a concomitant respiratory allergy—i.e., allergic rhinitis, or allergic rhinitis and asthma—excluding food allergies. Median sIgE and IQR values are shown. The number (%) of subjects (*n* = 36) sensitized to the corresponding mite molecular allergen is shown.

Mite Allergen	Median sIgE M (IQR)	No. of Sensitized Patients (%)
Der f 2	17.41 (37.21)	32 (88.88)
Der p 2	24.19 (41.42)	31 (86.11)
Der p 1	9.46 (27.31)	30 (83.33)
Der p 23	7.44 (24.89)	28 (77.77)
Der f 1	2.47 (9.95)	25 (69.44)
Der p 5	3.45 (27.02)	22 (61.11)
Der p 7	2.53 (25.03)	21 (58.33)
Blo t 21	0.2 (10.64)	19 (52.77)
Gly d 2	0.11 (3.71)	17 (47.22)
Tyr p 2	0.1 (1.75)	15 (41.66)
Der p 21	0.1 (9.83)	14 (38.88)
Blo t 5	0.1 (6.84)	14 (38.88)
Lep d 2	0.1 (2.19)	13 (36.11)
Der p 20	0.1 (0.0)	3 (8.33)
Der p 10	0.1 (0.0)	1 (2.77)
Blo t 10	0.1 (0.0)	2 (5.55)
Der p 11	0.1 (0.0)	0 (0)

## Data Availability

The data that support the findings of this study are available from the Servicio Canario de Salud but restrictions apply to the availability of these data, which were used under license for the current study, and so are not publicly available. Data are, however, available from the authors upon reasonable request and with the permission of the Servicio Canario de Salud.

## Abbreviations

The following abbreviations are used in this manuscript:

Tm	*Tenebrio molitor*
EIs	Edible Insects
EFSA	European Food Safety Authority
HDMs	House Dust Mites
TM	Tropomyosin
Lm	*Locusta migratoria*
Ad	*Acheta domesticus*
AK	Arginine Kinase

## Appendix A

**Table A1:** Correlations among each insect reactivity and recognition of 17 mite molecules, and their corresponding significances. Statistical significance was set at a *p*-value < 0.05.

Mite Allergen	*Acheta domesticus* r (*p* Value)	*Locusta migratoria* r (*p* Value)	*Tenebrio mollitor* r (*p* Value)
Der f 2	0.13 (0.1)	0.1 (0.21)	0.02 (0.79)
Der p 2	−0.28 (0.06)	−0.05 (0.34)	−0.15 (0.3)
Der p 1	0.32 (0.05)	0.29 (0.08)	0.14 (0.09)
Der p 23	0.28 (0.09)	0.25 (0.13)	0.24 (0.1)
Der f 1	0.18 (0.27)	0.13 (0.43)	0.03 (0.8)
Der p 5	0.25 (0.12)	0.29 (0.08)	0.17 (0.31)
Der p 7	0.24 (0.1)	0.21 (0.12)	0.22 (0.2)
Blo t 21	0.22 (0.18)	0.14 (0.39)	0.06 (0.69)
Gly d 2	0.06 (0.68)	−0.05 (0.72)	0.003 (0.98)
Tyr p 2	0.12 (0.09)	0.15 (0.1)	0.23 (0.16)
Der p 21	−0.07 (0.66)	−0.11 (0.51)	−0.15 (0.37)
Blo t 5	0.16 (0.33)	0.09 (0.58)	0.02 (0.88)
Lep d 2	0.23 (0.15)	0.09 (0.56)	0.18 (0.28)
Der p 20	0.29 (0.001)	0.31 (0.0019)	0.26 (0.0017)
Der p 10	0.71 (<0.0001)	0.77 (<0.0001)	0.82 (<0.0001)
Blo t 10	0.7 (<0.0001)	0.71 (<0.0001)	0.78 (<0.0001)
Der p 11	0.0 (0.0)	0.0 (0.0)	0.0 (0.0)

## References

[1] Commission Implementing Regulation (Eu) 2025/89 of 20 January 2025. https://eur-lex.europa.eu/legal-content/EN/TXT/PDF/?uri=OJ:L_202500089.

[2] Edible Insects: The Science of Novel Food Evaluations. https://www.efsa.europa.eu/en/news/edible-insects-science-novel-food-evaluations.

[3] Francis F., Doyen V., Debaugnies F., Mazzucchelli G., Caparros R., Alabi T., Blecker C., Haubruge E., Corazza F. (2019). Limited cross reactivity among arginine kinase allergens from mealworm and cricket edible insects. Food Chem..

[4] Barre A., Pichereaux C., Simplicien M., Burlet-Schiltz O., Benoist H., Rougé P. (2021). A Proteomic- and Bioinformatic-Based Identification of Specific Allergens from Edible Insects: Probes for Future Detection as Food Ingredients. Foods.

[5] de Gier S., Verhoeckx K. (2018). Insect (food) allergy and allergens. Mol. Immunol..

[6] Lamberti C., Nebbia S., Cirrincione S., Brussino L., Giorgis V., Romito A., Marchese C., Manfredi M., Marengo E., Giuffrida M.G. (2021). Thermal processing of insect allergens and IgE cross-recognition in Italian patients allergic to shrimp, house dust mite and mealworm. Food Res. Int..

[7] Jeong K.Y., Park J.W. (2020). Insect Allergens on the Dining Table. Curr. Protein Pept. Sci..

[8] Skotnicka M., Karwowska K., Kłobukowski F., Borkowska A., Pieszko M. (2021). Possibilities of the Development of Edible Insect-Based Foods in Europe. Foods.

[9] Barre A., Pichereaux C., Velazquez E., Maudouit A., Simplicien M., Garnier L., Bienvenu F., Bienvenu J., Burlet-Schiltz O., Auriol C. (2019). Insights into the Allergenic Potential of the Edible Yellow Mealworm (*Tenebrio molitor*). Foods.

[10] van Broekhoven S., Bastiaan-Net S., de Jong N.W., Wichers H.J. (2016). Influence of processing and in vitro digestion on the allergic cross-reactivity of three mealworm species. Food Chem..

[11] Sokol W.N. (2020). Grasshopper sensitization in patients allergic to crustaceans, mites, and cockroaches: Should grasshopper-containing products carry a warning?. Ann. Allergy Asthma Immunol..

[12] Purohit A., Shao J., Degreef J.M., van Leeuwen A., van Ree R., Pauli G., de Blay F. (2007). Role of tropomyosin as a cross-reacting allergen in sensitization to cockroach in patients from Martinique (French Caribbean island) with a respiratory allergy to mite and a food allergy to crab and shrimp. Eur. Ann. Allergy Clin. Immunol..

[13] Ribeiro J.C., Cunha L.M., Sousa-Pinto B., Fonseca J. (2017). Allergic risks of consuming edible insects: A systematic review. Mol. Nutr. Food Res..

[14] Sozener Z.C., Ozturk B.O., Cerci P., Turk M., Akin B.G., Akdis M., Altiner S., Ozbey U., Ogulur I., Mitamura Y. (2022). Epithelial barrier hypothesis: Effect of the external exposome on the microbiome and epithelial barriers in allergic disease. Allergy.

[15] Caraballo L., Zakzuk J., Lee B.W., Acevedo N., Soh J.Y., Sánchez-Borges M., Hossny E., García E., Rosario N., Ansotegui I. (2016). Particularities of allergy in the Tropics. World Allergy Organ. J..

[16] Muddaluru V., Valenta R., Vrtala S., Schlederer T., Hindley J., Hickey P., Larché M., Tonti E. (2021). Comparison of house dust mite sensitization profiles in allergic adults from Canada, Europe, South Africa and USA. Allergy.

[17] González-Pérez R., Galván-Calle C.A., Galán T., Poza-Guedes P., Sánchez-Machín I., Enrique-Calderón O.M., Pineda F. (2024). Molecular Signatures of Aeroallergen Sensitization in Respiratory Allergy: A Comparative Study Across Climate-Matched Populations. Int. J. Mol. Sci..

[18] Goodess C.M., Giorgi F., Hamaoui-Laguel L., Semenov M.A., Solmon F., Storkey J., Vautard R., Epstein M.M. (2017). Climate Change and Future Pollen Allergy in Europe. Environ. Heal. Perspect..

[19] Beggs P.J., Clot B., Sofiev M., Johnston F.H. (2023). Climate change, airborne allergens, and three translational mitigation approaches. eBioMedicine.

[20] Plume Labs. https://plumelabs.com/en/air/.

[21] Peel M.C., Finlayson B.L., McMahon T.A. (2007). Updated world map of the Köppen-Geiger climate classification. Hydrol. Earth Syst. Sci..

[22] Ansotegui I.J., Melioli G., Canonica G.W., Caraballo L., Villa E., Ebisawa M., Passalacqua G., Savi E., Ebo D., Gómez R.M. (2020). IgE allergy diagnostics and other relevant tests in allergy, a World Allergy Organization position paper. World Allergy Organ. J..

[23] Santos A.F., Riggioni C., Agache I., Akdis C.A., Akdis M., Alvarez-Perea A., Alvaro-Lozano M., Ballmer-Weber B., Barni S., Beyer K. (2023). EAACI guidelines on the diagnosis of IgE-mediated food allergy. Allergy.

[24] Kleine-Tebbe J., Jakob T. (2015). Molecular allergy diagnostics using IgE singleplex determinations: Methodological and practical considerations for use in clinical routine: Part 18 of the Series Molecular Allergology. Allergo J. Int..

[25] Kleine-Tebbe J., Jappe U. (2017). Molecular allergy diagnostic tests: Development and relevance in clinical practice. Allergologie.

[26] Platteel A.C., van der Pol P., Murk J.-L., Verbrugge-Bakker I., Hack-Steemers M., Roovers T.H., Heron M. (2022). A comprehensive comparison between ISAC and ALEX2 multiplex test systems. Clin. Chem. Lab. Med..

[27] González-Pérez R., Poza-Guedes P., Pineda F., Galán T., Mederos-Luis E., Abel-Fernández E., Martínez M.J., Sánchez-Machín I. (2023). Molecular Mapping of Allergen Exposome among Different Atopic Phenotypes. Int. J. Mol. Sci..

[28] Soil-Transmitted Helminthiases: Eliminating Soil-Transmitted Helminthiases as a Public Health Problem in Children: Progress Report 2001–2010 and Strategic Plan 2011–2020. https://apps.who.int/iris/bitstream/handle/10665/44804/9789241503129_eng.pdf.

[29] Bousquet J., Schünemann H.J., Togias A., Bachert C., Erhola M., Hellings P.W., Klimek L., Pfaar O., Wallace D., Ansotegui I. (2020). Allergic Rhinitis and Its Impact on Asthma Working Group. Next-generation Allergic Rhinitis and Its Impact on Asthma (ARIA) guidelines for allergic rhinitis based on Grading of Recommendations Assessment, Development and Evaluation (GRADE) and real-world evidence. J. Allergy Clin. Immunol..

[30] 2022 GINA Main Report. https://ginasthma.org/gina-reports/.

[31] Bojcukova J., Vlas T., Forstenlechner P., Panzner P. (2019). Comparison of two multiplex arrays in the diagnostics of allergy. Clin. Transl. Allergy.

[32] Lis K., Bartuzi Z. (2023). Selected Technical Aspects of Molecular Allergy Diagnostics. Curr. Issues Mol. Biol..

[33] Nösslinger H., Mair E., Oostingh G.J., Ahlgrimm-Siess V., Ringauf A., Lang R. (2024). Multiplex Assays in Allergy Diagnosis: Allergy Explorer 2 versus ImmunoCAP ISAC E112i. Diagnostics.

[34] Altmann F. (2016). Coping with cross-reactive carbohydrate determinants in allergy diagnosis. Allergo J. Int..

[35] Chen H., Jiang Q., Yang Y., Zhang W., Yang L., Zhu R. (2022). Cross-Reacting Carbohydrate Determinants Inhibitor Can Improve the Diagnostic Accuracy in Pollen and Food Allergy. J. Asthma Allergy.

[36] Omuse E.R., Tonnang H.E.Z., Yusuf A.A., Machekano H., Egonyu J.P., Kimathi E., Mohamed S.F., Kassie M., Subramanian S., Onditi J. (2024). The global atlas of edible insects: Analysis of diversity and commonality contributing to food systems and sustainability. Sci. Rep..

[37] Siddiqui S.A., Tettey E., Yunusa B.M., Ngah N., Debrah S.K., Yang X., Fernando I., Povetkin S.N., Shah M.A. (2023). Legal situation and consumer acceptance of insects being eaten as human food in different nations across the world-A comprehensive review. Compr. Rev. Food Sci. Food Saf..

[38] Sokol W.N., Wünschmann S., Agah S. (2017). Grasshopper anaphylaxis in patients allergic to dust mite, cockroach, and crustaceans: Is tropomyosin the cause?. Ann. Allergy Asthma Immunol..

[39] Ayuso R., Reese G., Leong-Kee S., Plante M., Lehrer S.B. (2002). Molecular basis of arthropod cross-reactivity: IgE-binding cross-reactive epitopes of shrimp, house dust mite and cockroach tropomyosins. Int. Arch. Allergy Immunol..

[40] Olivieri B., Stoenchev K.V., Skypala I.J. (2022). Anaphylaxis across Europe: Are pollen food syndrome and lipid transfer protein allergy so far apart?. Curr. Opin. Allergy Clin. Immunol..

[41] Betancor D., Gomez-Lopez A., Villalobos-Vilda C., Nuñez-Borque E., Fernández-Bravo S., De Las Heras Gozalo M., Pastor-Vargas C., Esteban V., Cuesta-Herranz J. (2021). LTP Allergy Follow-Up Study: Development of Allergy to New Plant Foods 10 Years Later. Nutrients.

[42] Mahammed L.L., Belaid B., Berkani L.M., Merah F., Rahali S.Y., Kaci A.A., Berkane I., Sayah W., Allam I., Djidjik R. (2022). Shrimp sensitization in house dust mite algerian allergic patients: A single center experience. World Allergy Organ. J..

[43] Farioli L., Losappio L.M., Giuffrida M.G., Pravettoni V., Micarelli G., Nichelatti M., Scibilia J., Mirone C., Cavallarin L., Lamberti C. (2017). Mite-Induced Asthma and IgE Levels to Shrimp, Mite, Tropomyosin, Arginine Kinase, and Der p 10 Are the Most Relevant Risk Factors for Challenge-Proven Shrimp Allergy. Int. Arch. Allergy Immunol..

[44] del Giudice M.M., Dinardo G., Klain A., D’addio E., Bencivenga C.L., Decimo F., Indolfi C. (2023). Anaphylaxis after Shrimp Intake in a European Pediatric Population: Role of Molecular Diagnostics and Implications for Novel Foods. Children.

[45] Giusti D., Guemari A., Perotin J.-M., Fontaine J.-F., Libyh M.T., Gatouillat G., Tabary T., Pham B.-N., Vitte J. (2024). Molecular allergology: A clinical laboratory tool for precision diagnosis, stratification and follow-up of allergic patients. Clin. Chem. Lab. Med..

[46] Riggioni C., Leung A.S., Wai C.Y., Davies J.M., Sompornrattanaphan M., Pacharn P., Chamani S., Brettig T., Peters R.L. (2025). Exploring geographical variances in component-resolved diagnosis within the Asia-Pacific region. Pediatr. Allergy Immunol..

[47] Mittermann I., Lupinek C., Wieser S., Aumayr M., Kuchler W.W., Chan A.W., Lee T.H., Zieglmayer P. (2022). IgE reactivity patterns in Asian and central European cockroach-sensitized patients reveal differences in primary sensitizing allergen sources. J. Allergy Clin. Immunol. Glob..

[48] Wangorsch A., Jamin A., Spiric J., Vieths S., Scheurer S., Mahler V., Hofmann S.C. (2024). Allergic Reaction to a Commercially Available Insect Snack Caused by House Cricket (*Acheta domesticus*) Tropomyosin. Mol. Nutr. Food Res..

[49] Li J.C., Rotter N.S., Stieb E.S., Stockbridge J.L., Theodorakakis M.D., Shreffler W.G. (2023). Utility of food allergy thresholds. Ann. Allergy Asthma Immunol..

[50] Valenta R., Karaulov A., Niederberger V., Gattinger P., van Hage M., Flicker S., Linhart B., Campana R., Focke-Tejkl M., Curin M. (2018). Molecular Aspects of Allergens and Allergy. Adv. Immunol..

[51] Sharma E., Vitte J. (2024). A systematic review of allergen cross-reactivity: Translating basic concepts into clinical relevance. J. Allergy Clin. Immunol. Glob..

[52] Dramburg S., Hilger C., Santos A.F., Vecillas L.d.L., Aalberse R.C., Acevedo N., Aglas L., Altmann F., Arruda K.L., Asero R. (2023). EAACI Molecular Allergology User’s Guide 2.0. Pediatr. Allergy Immunol..

[53] De Marchi L., Wangorsch A., Zoccatelli G. (2021). Allergens from Edible Insects: Cross-reactivity and Effects of Processing. Curr. Allergy Asthma Rep..

[54] Scala E., Abeni D., Villella V., ViIlalta D., Cecchi L., Caprini E., Asero R. (2024). Investigating Novel Food Sensitization: A Real-Life Prevalence Study of Cricket, Locust, and Mealworm IgE-Reactivity in Naïve allergic Individuals. J. Investig. Allergol. Clin. Immunol..

[55] Wong L., Huang C.H., Lee B.W. (2016). Shellfish and House Dust Mite Allergies: Is the Link Tropomyosin?. Allergy Asthma Immunol. Res..

[56] Emilia M., Magdalena C., Weronika G., Julia W., Danuta K., Jakub S., Bożena C., Krzysztof K. (2025). IgE-based analysis of sensitization and cross-reactivity to yellow mealworm and edible insect allergens before their widespread dietary introduction. Sci. Rep..

[57] Linacero R., Cuadrado C. (2022). New Research in Food Allergen Detection. Foods.

[58] Retzinger A.C., Retzinger G.S. (2021). The Acari Hypothesis, II: Interspecies Operability of Pattern Recognition Receptors. Pathogens.

[59] Trompette A., Divanovic S., Visintin A., Blanchard C., Hegde R.S., Madan R., Thorne P.S., Wills-Karp M., Gioannini T.L., Weiss J.P. (2009). Allergenicity resulting from functional mimicry of a Toll-like receptor complex protein. Nature.

[60] Mueller G.A., Edwards L.L., Aloor J.J., Fessler M.B., Glesner J., Pomés A., Chapman M.D., London R.E., Pedersen L.C. (2010). The structure of the dust mite allergen Der p 7 reveals similarities to innate immune proteins. J. Allergy Clin. Immunol..

[61] Pali-Schöll I., Meinlschmidt P., Larenas-Linnemann D., Purschke B., Hofstetter G., Rodríguez-Monroy F.A., Einhorn L., Mothes-Luksch N., Jensen-Jarolim E., Jäger H. (2019). Edible insects: Cross-recognition of IgE from crustacean- and house dust mite allergic patients, and reduction of allergenicity by food processing. World Allergy Organ. J..

[62] Popescu F.D. (2015). Cross-reactivity between aeroallergens and food allergens. World J. Methodol..

[63] Shroba J., Rath N., Barnes C. (2018). Possible Role of Environmental Factors in the Development of Food Allergies. Clin. Rev. Allergy Immunol..

[64] Belluco S., Losasso C., Maggioletti M., Alonzi C.C., Paoletti M.G., Ricci A. (2013). Edible Insects in a Food Safety and Nutritional Perspective: A Critical Review. Compr. Rev. Food Sci. Food Saf..

[65] Conway A., Jaiswal S., Jaiswal A.K. (2024). The Potential of Edible Insects as a Safe, Palatable, and Sustainable Food Source in the European Union. Foods.

[66] Zhao L., Zhang Y., Zhang S., Zhang L., Lan F. (2021). The effect of immunotherapy on cross-reactivity between house dust mite and other allergens in house dust mite -sensitized patients with allergic rhinitis. Expert Rev. Clin. Immunol..

[67] Verhoeckx K.C., van Broekhoven S., den Hartog-Jager C.F., Gaspari M., de Jong G.A., Wichers H.J., van Hoffen E., Houben G.F., Knulst A.C. (2014). House dust mite (Der p 10) and crustacean allergic patients may react to food containing Yellow mealworm proteins. Food Chem. Toxicol..

[68] Broekman H.C.H.P., Knulst A.C., de Jong G., Gaspari M., Jager C.F.D.H., Houben G.F., Verhoeckx K.C.M. (2017). Is mealworm or shrimp allergy indicative for food allergy to insects?. Mol. Nutr. Food Res..

[69] Gałęcki R., Bakuła T., Gołaszewski J. (2023). Foodborne Diseases in the Edible Insect Industry in Europe-New Challenges and Old Problems. Foods.

[70] Abro Z., Sibhatu K.T., Fetene G.M., Alemu M.H., Tanga C.M., Sevgan S., Kassie M. (2025). Global review of consumer preferences and willingness to pay for edible insects and derived products. Glob. Food Secur..

[71] Quintieri L., Nitride C., De Angelis E., Lamonaca A., Pilolli R., Russo F., Monaci L. (2023). Alternative Protein Sources and Novel Foods: Benefits, Food Applications and Safety Issues. Nutrients.

[72] Sicherer S.H., Sampson H.A. (2010). Food allergy. J. Allergy Clin. Immunol..

[73] Schussler E., Kattan J. (2015). Allergen Component Testing in the Diagnosis of Food Allergy. Curr. Allergy Asthma Rep..

